# Acute and Chronic Effects of Crude Oil Water-Accommodated Fractions on the Early Life Stages of Marine Medaka (*Oryzias melastigma*, McClelland, 1839)

**DOI:** 10.3390/toxics11030236

**Published:** 2023-02-28

**Authors:** Fei Jin, Ying Wang, Fuwei Yu, Xing Liu, Mingxing Zhang, Zhaochuan Li, Ziwei Yao, Yi Cong, Juying Wang

**Affiliations:** 1 Key Laboratory for Ecological Environment in Coastal Areas, National Marine Environmental Monitoring Center, No. 42 Linghe Street, Dalian 116023, China; 2 School of Chemical Engineering, Dalian University of Technology, Dalian 116023, China

**Keywords:** water-accommodated fractions, *Oryzias melastigma*, early-life stage, mortality, histopathology, hatch, development

## Abstract

Oil spill is a major marine environmental pollution issue. Research regarding the long-term effects of oil spills on the early life stage of marine fish is still limited. In this study, the potential adverse impact of crude oil from one oil spill accident which occurred in the Bohai Sea on the early life stages of marine medaka (*Oryzias melastigma*, McClelland, 1839) was evaluated. A 96-h acute test (larvae) and a 21-d chronic test (embryo–larvae) of water-accommodated fractions (WAFs) from crude oil were conducted, respectively. The results of the acute test showed that only the highest concentration of WAFs (100.00%) significantly affected the mortality of larvae (*p* < 0.01) and that the 96 h-LC_50_ was 68.92% (4.11 mg·L^-1^ expressed as total petroleum hydrocarbons (TPHs)). Larval heart demonstrated histopathological alterations in all WAF-exposed groups. The chronic test results showed that, except for larval mortality, the total hatching success (%)/hatching time of embryos in WAF treatments was not significantly different from those of the control group (*p* > 0.05), and no malformation was found in surviving larvae after 21 d of exposure. Nevertheless, the exposed embryos and larvae in the highest concentration of WAFs (60.00%) demonstrated significantly reduced heart rate (*p* < 0.05) and increased mortality (*p* < 0.01), respectively. Overall, our results indicated that both acute and chronic WAF exposures had adverse impacts on the survival of marine medaka. In the early life stages, the heart of the marine medaka was the most sensitive organ which showed both structural alteration and cardiac dysfunction.

## 1. Introduction

Crude oil is a mixture composed of quantities of saturated and aromatic hydrocarbons, as well as a few of nonhydrocarbon components, such as colloid and bitumen, which belong to an unknown or variable composition, complex reaction products, or biological materials (UVCBs). Crude oil and gas exploration, production, and transport activities present a risk of accidental oil spills with potential consequences for marine ecosystems. The chemical composition of crude oils from different places of production varies greatly [1,2] and the bioavailability of crude oil plays an important role in its toxicity. Currently, it is thought that the water-accommodated fraction (WAF) or water-soluble fraction (WSF) of crude oil and its derivative products are the main components possibly responsible for its observed toxicities [3]. Of which, those causing the most concern regarding environmental risk are the polycyclic aromatic hydrocarbons (PAHs), as they exhibit high toxicity in aquatic environments [4,5].

The early life stage of fish is considered to be the most sensitive life stage during its lifecycle [6,7], and both the embryonic and larval stages are commonly selected in (eco)toxicological studies of crude oils. Previous studies have showed that exposures of crude oils have resulted in considerably negative effects on the early life stages of fishes at different biological levels. Besides mortality observed in fish such as *Anchoa mitchilli* [8], other toxicological consequences of crude oils to marine fishes have been revealed, including growth retardation [8], developmental retardation of the brain and abnormality of swimming behavior of *Takifugu rubripes* [9], genotoxicity and histopathological damage of *Centropomus parallelus* [10], as well as gene expression alteration related to lipid metabolism in *Coryphaena hippurus* [11]. Among the influences of crude oils on the early life stage of fishes, survival and development are ecologically relevant endpoints, which are critical in ecotoxicological studies of crude oils. Histopathological biomarkers have been widely used in environmental monitoring, as these allow for an examination of specific target organs such as gills, liver, and so on, that are responsible for vital functions such as excretion and the biotransformation of xenobiotics in the fish. There are increasing reports about histological alterations in fish gills or livers due to the toxicity of spilt oil [12,13] or in the laboratory exposures of fish to petroleum oil [14,15]. However, the histopathological study of other potential target organs in fish including heart, brain, and intestine after oil exposure [6] is still limited.

After oil spills, the pollution usually lasts for a long time, and short-term assessment cannot truly reflect the long-term effects of oil spills. Among a few long-term exposure studies on the early life stage of fishes, due to the acclimatation period in labs after the collection of fish embryos from the field, it is impossible to carry out experiments within a few hours after fertilization [16,17,18], which makes it difficult to accurately assess the impact of oil spill on the intact development stage of fish embryos. Therefore, studies on the long-term toxic effects of crude oil using the early life stages, especially the stage immediately after fertilization, of small marine model fishes are still urgently needed. Marine medaka, *Oryzias melastigma* (McClelland, 1839), with characteristics including a small individual size, short generation cycle, easy to distinguish gender, ability to reproduce all year round, easy to observe the early life stages and to cultivate in the laboratory, a wide range of temperature and salinity adaptations, and sensitivity to the pollutants, is an ideal marine model organism for ecotoxicology and environmental studies in Asian regions [19]. It has been recommended by the Health and Environmental Science Institute (HESI) to be adopted for embryo toxicity tests [20], which provides an effective biological tool for assessing the acute and chronic toxic effects of pollutants. In this study, the impacts of crude oil from the Bohai oil spill accident on the early life stages (embryos and larvae) of marine medaka were assessed using endpoints with ecological relevance (mortality, hatching, and malformation) through a 96-h acute test and a 21-d chronic test, respectively, with the expection of providing a scientific basis for ecological risk assessment after oil spill pollution.

## 2. Materials and Methods

### 2.1. Test Organism

Marine medaka, *O. melastigma* (McClelland, 1839), was cultured using a flowing circulation system (Stand-Alone System AH2030, Aquatic Habitats^TM^, USA) in our laboratory for more than 15 years. The culturing artificial seawater (<0.45 μm, also used for exposure experiments) was prepared with coral reef sea salts purchased from Engineering Technology Institute Co., LTD of CNSIC (Tianjin, China) and reverse osmosis water. The culture conditions were as follows: temperature 27 ± 1 °C, salinity 32 ± 1, pH 8.0 ± 0.1, light dark ratio of 14 h:10 h. All the medaka juveniles and adults were fed 3 times per day with *Artemia* nauplii, which were harvested from frozen eggs (Haiyou Jiayin Biotechnology Co., Ltd., Tianjin, China) incubated in artificial seawater for 24 h, and larval fishes were fed twice per day with Marubeni Nissin C1 fish food (Marubeni Nisshin Feed Co., Ltd., Tianjin, China). Medaka embryos were collected daily, washed with clean artificial seawater, and incubated in the system.

### 2.2. Preparation of Water-Accommodated Fraction (WAF) of Crude Oil

The crude oil sample used in this study was donated by a corporation in China. The preparation of WAFs from crude oil was based on a gravimetric method according to the industry standard (HY/T 256-2018) and Barron and Ka’aihue [21], with some modifications. In brief, one hundred grams of crude oil was added to 4 L of artificial seawater (32 ± 1, <0.45 μm), leaving 20% ~ 30% of the total solution volume between the liquid level and the mouth of the tap bottle. The mixed solution was stirred with a magnetic stirrer (C-MAG HS 7, Ika Company, Staufen, Germany) under sealed conditions, and the depth of the vortex was about 20% ~ 25% of the depth of the solution. After stirring continuously for 18 h and standing for 6 h, the lower aqueous phase was collected from the bottom mouth of the tap bottle as a WAF stock solution, which was prepared in three replicates and mixed as a preservation solution in a brown bottle stored at 4 °C. The different exposure concentrations of WAF in the following toxicity tests were prepared by diluting the stock WAF solution with different dilution factors.

### 2.3. Total Petroleum Hydrocarbon (TPH) and PAH Analyses

The concentrations of total petroleum hydrocarbons (TPHs) in exposure media were determined using UV spectrophotometry. The standard petroleum substance (No. GBW(E)080913, 1000 mg⋅L^–1^, prepared by National Marine Environmental Monitoring Center) was diluted with n-hexane (chromatographic pure, Thermo Fisher, Waltham, MA, USA) to a constant volume, and its absorbance at 255 nm was measured using a ultraviolet spectrophotometer (UV5Nano, Mettler Toledo, Switzerland) followed by the drawing of the standard curve. The exposure solutions were acidified with sulfuric acid and extracted with n-hexane twice. The absorbance of the extraction solution was measured at 255 nm, and the TPH concentrations of the exposure solutions were calculated according to the standard curve (3 replicates for each concentration).

The concentrations of 16 priority PAHs listed by the US Environmental Protection Agency (EPA) were measured using gas chromatography–mass spectrometry (GC–MS) following the method of Mu et al. [22]. Briefly, 50 mL of a WAF sample was extracted and added to 40 mL of dichloromethane after spiking with a mixture of internal standards (2-Fluorobiphenyl, *p*-Terphenyl-*d*_14_). Each extract was concentrated using a rotary evaporator, exchanged into n-hexane, and spiked with injection standards (naphthalene-*d*_8_, acenaphthene-*d*_10_, phenanthrene-*d*_10_, chrysene-*d*_12_, and perylene-*d*_12_). Extracts were analyzed with an Agilent 6890 GC coupled to a 5975 MS in the selective ion-monitoring mode. Recoveries of 2-Fluorobiphenyl and *p*-Terphenyl-*d*_14_ surrogate standards were 62.85 ± 1.27% (mean ± standard deviation) and 76.61 ± 1.12%, respectively.

### 2.4. Acute Toxicity Test

According to the results of the pilot study, the nominal concentrations of the WAF in the acute toxicity test were set as 9.53%, 17.15%, 30.86%, 55.56%, and 100.00% (the dilution factor is 1.8), respectively, with artificial seawater as the blank control. The control group and each treatment contained 3 replicates. The exposure containers were 20 mL glass scintillation bottles, each containing 10 mL of the corresponding concentration of the exposure solution and 10 randomly assigned healthy larvae (1 dph, days post hatching). All groups were incubated in a constant temperature illumination incubator (Zhichu Instrument Co., LTD., ZQLY-180G, Shanghai, China) at 27 ± 1 °C under a cycle of 14 h illumination/10 h dark for 96 h, and the exposure solutions were replaced once every 24 h. No food was supplied during exposure. At 24 h, 48 h, 72 h, and 96 h, the mortality of larvae in the control and treated groups was recorded. If there is no reaction for a long time and no respiration and heartbeat can be observed under an inverted microscope (Leica DMI4000B, Germany), the larva is determined to be dead.

### 2.5. Chronic Toxicity Test

The chronic toxicity test was conducted for 21 d during the early life stages of marine medaka, covering their whole embryo stage and a part of the larval stage. Three nominal exposure concentrations of WAFs (15%, 30%, and 60%) were selected in the chronic toxicity test according to the result of the acute toxicity test. The exposure setup was similar to the acute toxicity test, with each glass scintillation bottle containing 10 mL of the solution and 10 randomly assigned healthy embryos (6 hpf, hours post fertilization). After hatching (medaka embryos usually begin to hatch at 12 dpf), the larvae were transferred to 10 mL of the corresponding concentration of solutions to continue exposure until 21 d (the age of larvae ranged from 1 dph to 8 dph at the end of 21-d exposure). All groups were incubated in an incubator at 80 rpm and 27 ± 1 °C under a cycle of 14 h illumination/10 h dark, and the exposure solutions were replaced once every 24 h. Medaka larvae were fed with Marubeni Nissin C1 fish food once a day.

### 2.6. Histopathological Analyses

After 96 h of exposure, six larvae from each group were randomly selected, completely fixed with Bouin’s solution for around 24 h, and preserved in 75% ethanol. Afterwards, the larvae were dehydrated with gradient ethanol and embedded with paraffin as follows: 80%, 90%, 95%, 100%, and 100% ethanol for 4 min, absolute ethanol-xylene (1:1) for 2.5 min, xylene for 1 min with two times, and 65 °C melting paraffin for 20 min and 40 min, respectively. Then, the paraffin-infiltrated larvae were embedded with melting paraffin and cooled at −20 °C for 30 min. After that, each paraffin block was cut with Slicer Pathology (Leica 2235, Shanghai) into slices of 8 μm thickness, and each slice was flattened on water at 40 °C, picked up using a glass slide, and dried at 60°C for 1 h. Then, the slides were deparaffinated with xylene, hydrated with gradient ethanol, stained with ematoxylin and eosin, dehydrated with gradient ethanol, and made transparent using xylene. The slides were preserved at room temperature, and histological images of the larval vertical sections were photographed using a panoramic slice scanner (3DHISTECH, PANNORAMIC DESK/MIDI/250/1000, Hungary).

### 2.7. Developmental Toxicities

The endpoints of the developmental toxicity test were embryonic mortality, heart rate, percent total hatching success (%) and hatching time, as well as larval mortality, malformation percentage (%), and malformation characteristics. At the 11 d of exposure, the embryos were observed under an inverted microscope (Leica DMI4000B), and their heart rates were recorded by counting their number of heart beats over a 30 s period (10 replicates). After 21 d of exposure, the unhatched embryos were considered as a hatching failure and were no longer observed. Mortality, percent total hatching success (%), and hatching time of embryos, as well as larval mortality, were calculated accordingly. Malformation assessment of larvae was conducted according to the method of Scott et al. (2009), and the development of the larval heart, pericardial sac, yolk sac, craniofacial, spine, and blood circulation were evaluated followed by the larval malformation percent (%) calculation.

### 2.8. Statistical Analyses

Statistical analyses were performed using SPSS 19.0 software (SPSS, Chicago, IL, USA). The normality of data and the homogeneity of variances were verified using the Kolmogorov–Smirnov test and Levene’s test, respectively. A one-way analysis of variance (ANOVA) or nonparametric test (Kruskal–Wallis) was applied to determine the significant differences between the treatments and the controls depending on the assumptions of the parametric tests fulfilled or not. Differences were considered significant at *p* < 0.05. The 96-h median lethal concentration (LC_50_) of acute toxicity was calculated using Karber’s method. No observed effect concentration (NOEC) was determined as the highest concentration without a significant effect observed, and the lowest observed effect concentration (LOEC) was considered as the lowest concentration with a significant effect observed.

## 3. Results

### 3.1. Concentrations of TPHs and PAHs in Exposure Media

Measured concentrations of TPHs in acute and chronic toxicity tests at the start of the experiments are shown in Table 1. There was a good linear correlation between the nominal concentration of the WAF and the measured concentration of TPHs, and the correlation coefficient was *r* = 0.997 (*p* < 0.01) for the acute toxicity test and *r* = 0.982 (*p* < 0.01) for the chronic toxicity test, respectively. The exposure concentrations are expressed as nominal concentrations of WAFs in the result and discussion section for the convenience of reader.

Table 2 shows the concentrations of the individual and total (Σ16PAHs) PAH concentrations analyzed in a WAF. The Σ16PAHs concentration in the WAF was 1.77 mg·L^−1^, of which fluorene, anthracene, and chrysene were the three most abundant PAHs, with concentrations being 0.29 ± 0.05, 0.31 ± 0.16, and 0.36 ± 0.03 mg·L^−1^, and accounted for more than 50% for Σ16PAHs. In accordance with the low water solubility of the high molecular weight PAHs, the concentrations of PAHs of more than four rings were, in general, below the detection limit.

### 3.2. Acute Toxicity

**Figure 1:**
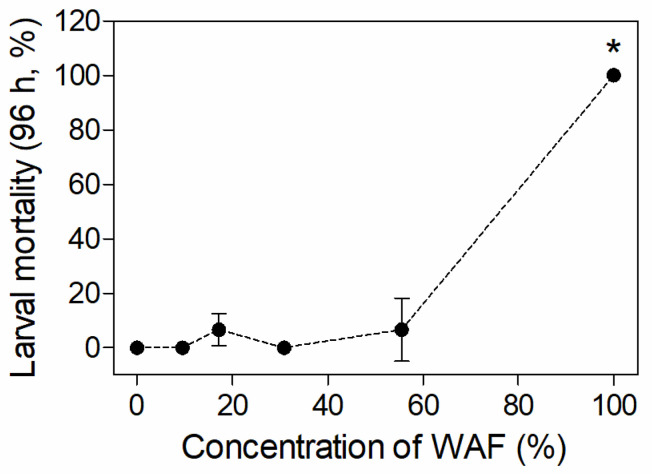
Mortality of marine medaka larvae after 96 h of exposure in acute test (*n* = 3). The symbol * indicates significant difference of the treatment compared to the control (*p* < 0.05).

As shown in Figure 1, no larval mortality was observed in the control and9.53%/30.86% WAF groups after 96 h of exposure, while the mortality in the 17.15% and 55.56% WAF treatments was 6.67%. However, the mortality in the 100% WAF treatment was up to 96.67% after 48 h, and reached 100% after 72 h. The statistical result indicates a significant increase in mortality in the 100% WAF treatment compared to the control (*p* < 0.05) after 96 h of exposure (*p* > 0.05). The 96 h-LC_50_ of the WAF of marine medaka larvae was 68.92% (TPHs as 4.11 mg·L^−1^), with a 95% confidence interval that ranged from 63.77% to 74.49% (TPHs as 3.77–4.48 mg·L^−1^).

### 3.3. Chronic Toxicity

#### 3.3.1. Mortalities of Embryos and Larvae

**Figure 2:**
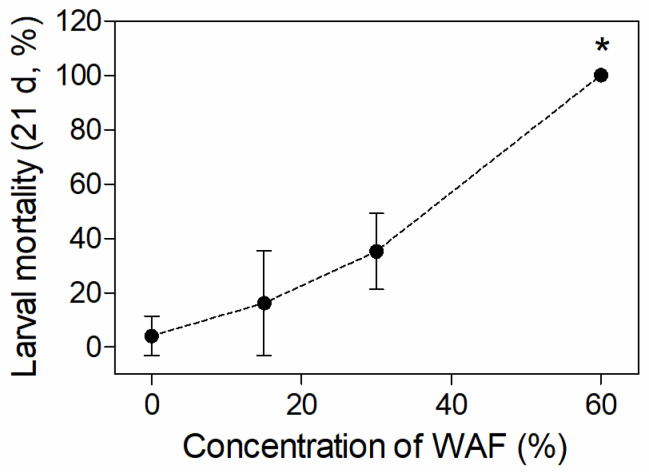
Mortality of marine medaka larvae after 21 d of exposure in chronic test (*n* = 3). The symbol * indicates significant difference of the treatment compared to the control (*p* < 0.05).

One dead embryo but no dead larva was found in the control group during the 21-d embryo and larvae exposure. The embryo mortalities in different WAF-treated groups were all higher than that of the control, but no significant difference of mortality (*p* > 0.05) was observed between the WAF treatments and the control (Table 3). The larval mortalities raised with increasing WAF concentrations, and the mortality in the 60% WAF treatment was significantly higher than those of the control (*p* < 0.01) (Figure 2).

#### 3.3.2. NOECs and LOECs

The NOECs and LOECs of embryonic mortality, heart rate, percent total hatching success (%), and hatching time, as well as larval mortality and malformation percent (%) after WAF treatment, are shown in Table 4. Of which, the NOECs and LOECs of the WAF of the embryo heart rate/larvae mortality was 30% (2.07 mg·L^−1^ as TPHs) and 60% (3.39 mg·L^−1^ as TPHs), respectively, while the NOECs of the other endpoints was 60% (3.39 mg·L^−1^ as TPHs).

#### 3.3.3. Histopathology

**Figure 3:**
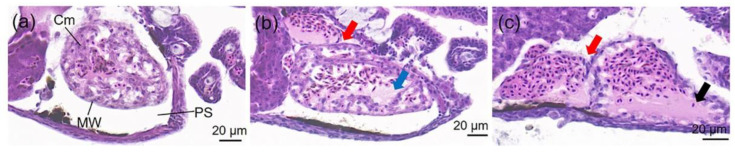
Histology (longitudinal section) of *O. melastigma* (McClelland, 1839) heart after 96 h of exposure to (**a**) control, (**b**) 9.53% WAF and (**c**) 55.56% WAF. PS: pericardial sac; Cm: cardiomyocyte; MW: myocardial wall. The typical histopathological alterations of heart were characterized with different symbols: cardiac malposition (red arrow), decrease in cardiomyocyte (blue arrow) and dilatation of heart (black arrow).

There was no histopathological change in the brain, heart, liver, and intestine in all individuals of the control groups. In the WAF-exposed groups, only the heart demonstrated histopathological alterations, and the percentage of individuals with alterations were 33.33%, 50%, 50%, and 83.33% in the 9.53%, 17.15%, 30.86%, and 55.56% WAF groups, respectively. The main histopathological alterations of the larval hearts included cardiac malposition (Figure 3b,c), decrease in cardiomyocyte (Figure 3b) and, dilatation of the heart (Figure 3c).

#### 3.3.4. Developmental Indicators

**Figure 4:**
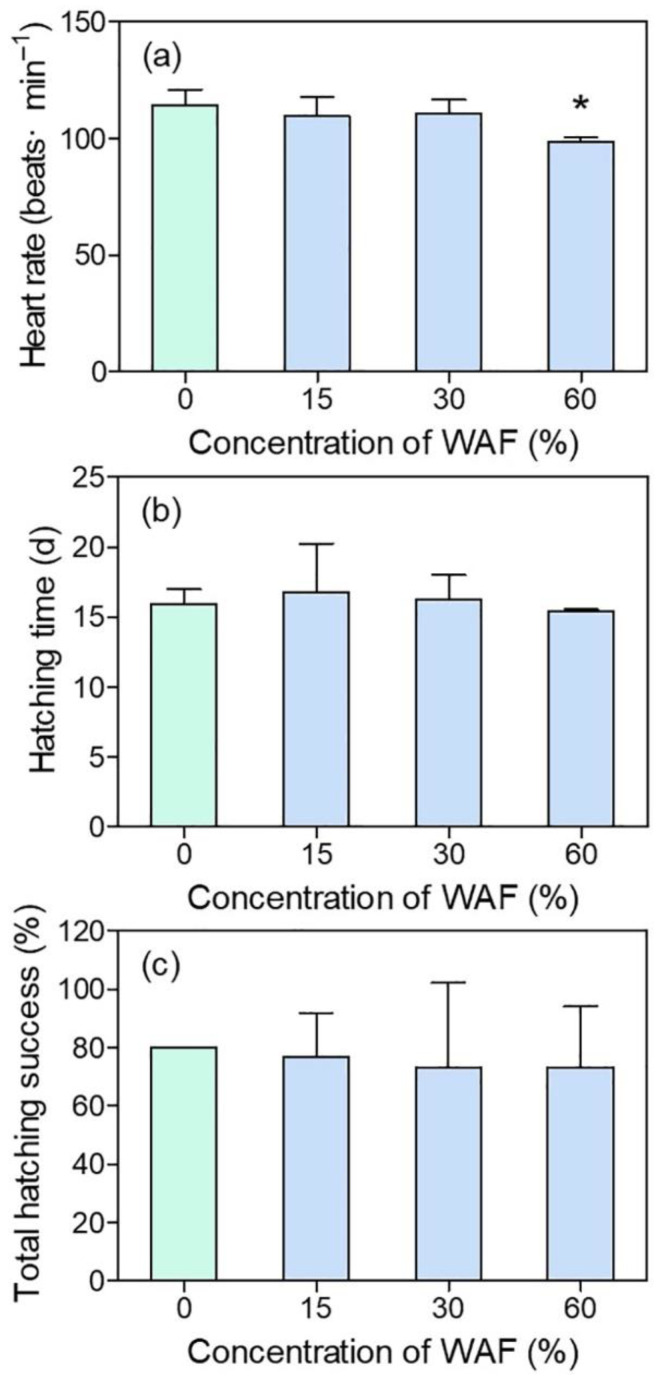
Heart rate (**a**), hatching time (**b**), and total hatching success (%) (**c**) of marine medaka embryos in chronic test (*n* = 3). The symbol * indicates significant difference of the treatment compared to the control (*p* < 0.05).

The average heart rate of marine medaka larvae in the control group at 11 d was 114 beats/min. The heart rate of WAF-treated embryos showed a general decrease along with WAF concentrations, especially in the 60% WAF-treated group, which was significantly lower than that of the control (*p* < 0.05) (Figure 4a). However, there was no significantly decreased heart rates in the 15% and 30% WAF treatments compared to the control (Figure 4a). For the hatching endpoints, hatching time and total hatching success (%) of embryos in the three WAF treatments showed no significant difference with those of the control (*p* > 0.05, Figure 4b,c), nor was any malformation of the WAF-treated larvae observed.

## 4. Discussion

### 4.1. Acute and Chronic Lethal Toxicities of WAF

Fish embryos and larvae are of particular concern after oil spill in part because they are not be able to avoid polluted areas. An investigation carried out in Alaska after the 1989 Exxon Valdez oil spill revealed that elevated mortality was observed in pink salmon (*Oncorhynchus gorbuscha*) embryos spawned in intertidal stretches of stream deltas several months after the spill. In this study, the mortalities of marine medaka embryos and larvae in the sensitive early life stages were assessed after 96-h and 21-d of exposures to the WAF of crude oil. As a consequence, the embryonic mortality was not significantly affected, while larval mortality was found only to be significantly affected at the highest concentration for both acute and chronic tests. The reason for the lower mortality observed in embryos should be attributed to the ptotective function of chorion, which decrease the entrance of pollutants by accumulating them on the surface of chorion [23]. In addition, the protective effect of embryonic chorions against pollutants has also been identified through the enhancement of sensitivity to exogenous compounds after chorion removal [24,25,26]. Thus, for medaka larvae without the protection of chorions after hatching, their mortalities tended to increase through direct interaction with toxic components in WAF, especially in the highest exposure groups, as observed in our study. This emphasizes the sensitivity difference of the embryonic and larval stages of marine medaka to crude oil within test concentrations. Moreover, although larval mortalities were assessed for both acute and chronic tests, the effect concentrations that differ significantly from the control in chronic test (60%) was lower than that in the acute test (100%). This possibly suggests that embryonic exposure to the WAF increases the sensitivity of hatched larvae, which, in turn, results in their increasing mortalities during chronic test.

Generally, unlike traditional single-component chemicals, the toxicological responses of organisms to pollutants with complex components such as crude oils do not necessarily change in a dose-dependent manner. In this study, as mentioned, we found that a WAF concentration of less than 55.6%/30% had no significant impact on the mortality of marine medaka larvae compared to the control, while the mortality increased rapidly to 100% at a WAF concentration of 100%/60% in the acute/chronic test. Rodrigues et al. [3] observed similar results in their study, namely, that 10% and 25% WSF did not cause the death of the newly hatched larvae of *Odontesthes argentinensis* after 96 h of exposure, while the mortality rate of larvae at 50% of WSF exposure was more than 50%. The above results demonstrated a non-dose-response relationship between mortality and WAF/WSF concentrations for marine fishes. Nevertheless, under some circumstances, marine fish mortalities were not influenced even at an exposure concentration of 100% WAF. For instance, in the study of Kim et al. [27], which adopted the same WAF preparation method and test species as in our study, no significant mortality of marine medaka adult fish (5 months old) was found, even for the 100% WAF concentration after 96 h of exposure. Likewise, there was no significant lethal effect of WAF on Gulf killifish within an experimental concentration range when the dissolved oxygen level was above 4 mg·L^−1^ [28]. The main reasons for these differences in the outcome of death from WAF/WSF exposure can possibly be attributed to the different life stages of test fishes, the different toxic components of various crude oils, and the different solubilities of the toxic components in crude oils. 

Considering the percentage concentration, the 96 h-LC_50_ of the WAF of the marine medaka larvae was calculated as 68.92% in this study. In some studies, the 96 h-LC_50_s of the WAF/WSF of the marine larval or juvenile fishes were close to our result. For instance, Rodrigues et al. [3] reported a 96 h-LC_50_ of 70.68% using WSF for *O. argentinensis*. Similarly, the 96 h-LC_50_s of the WAF of two-week old larvae of *Siganus canaliculatus* [15] and *Epinephelus chlorostigma* [29] were 70% and 75%, respectively. By contrast, in some studies, the 96 h-LC_50_ of WSF to marine larval or juvenile fish was quite different to study, such as, for example, the 37.15% of WSF to *Mugil liza* reported by Moreira et al. [30]. Nevertheless, although the percentage concentration was used in all of the above studies, it should be noted that the compositions and contents of toxic components in exposure solutions were greatly different due to the differences in crude oil composition and in the WAF/WSF preparation methods [31], which results in incomparable toxicity outcomes among the above studies. Therefore, the characterization of crude oil toxicity using mass concentration would be more pertinent, instead of using the percentage concentration of toxic components, such as the TPHs, total hydrocarbon content (THC) [32], total PAHs (TPAH) [33,34], total volatile organic analytes (VOAs) [35], and even the mass of oil dispersion [36]. In this study, a total TPH concentration of 100% WAF was 6.141 ± 0.037 mg·L^−1^, which was comparable to those of, for instance, Cohen et al. [37], Beirão et al. [34], and Gusmão et al. [38], in which the TPH concentrations of WAF/WSF were prepared using the same method, and was 2.7 mg·L^−1^, 1.85 mg·L^−1^, and 4.86 mg·L^−1^, respectively. Considering the TPH concentration of the WAF, the 96 h-LC_50_ of the WAF to marine medaka larvae was calculated as 4.11 mg·L^−1^ in our study. This result is similar to that of Barron et al. [32], in which the 96-h LC_50_s of three crude oils, represented as TPHs to silverside (*M. beryllina*), reportedly ranged from >9.93 mg·L^−1^ to >13 mg·L^−1^. Moreover, we note that, if expressed as the TPAH concentration, the LC_50_s of crude oil for fishes are usually lower than those expressed as the TPH concentration by one order of magnitude, as demonstrated by Duffy et al. [8] and Barron et al. [32]. This suggests that PAHs in the WAF are possibly the main components responsible for the toxicity of crude oils. However, for chronic exposure, due to a lack of information on the chronic lethal toxicity of WAF expressed as LOEC/NOEC to marine fishes, a further comparison of our results to the literature was infeasible at present. Nevertheless, we compared our chronic effect concentrations of TPHs with the “Sea Water Quality Standard (GB 3097-1997)” of China, in which the standard of the petroleum pollutant in seawater ranged from 0.05 to 0.5 mg·L^−1^ for Class I to Class IV water quality. The lowest NOEC of the WAF in our chronic test was 2.07 mg·L^−1^, expressed as TPHs, which is higher than that of GB 3097-1997 by one to two orders of magnitude. It seems that the current seawater quality standard in China can provide sufficient protection of marine fish from the influence of a petroleum pollutant. However, it should be noted that we conducted a partial early life chronic test with marine medaka, the NOEC/LOEC results of which cannot represent the whole early life chronic test, which could be even more lower. Therefore, more chronic tests covering the whole sensitive early life stages of more representative model marine fishes, together with a real-time field survey of crude oil concentrations in oil spill spots, should be performed before a definite conclusion can be conculded regarding the ecological risk of oil spill. Therefore, we would state that this study provides important information in favor of the risk assessments of crude oil WAF in marine environments, especially for its long-term influence.

### 4.2. Histopathological Alterations

In this study, the whole longitudinal section of marine medaka larvae were analyzed for histopathological alterations after the 96 h of exposure to the WAF of crude oil. Nevertheless, of the body parts studied, the heart was found to be the only sensitive organ with histopathological alterations, mainly including cardiac malposition (the atria misaligned with the ventricles), a decrease in cardiomyocyte, and dilatation of the heart (Figure 3), which indicated that the function of the circulation system in medaka larvae was potentially influenced after exposure to WAF.

Previous histopathological results with marine fishes had proved that the gill and liver, which are involved in fish physiological processes such as respiration, osmoregulation, acid-base balance, nitrogenous waste excretion, and xenobiotic metabolism, as well as detoxification, are the major organs after exposure to the WAF/WSF of crude oils, as demonstrated in the studies of Agamy et al. [14,15,29] and Moreira et al. [30]. A limited number of studies have showed that the fish heart was capable of demonstrating histopathological lesions mainly related to the morphology of ventricles. For instance, Gardner et al. (2019) reported that cardiac ventricles in exposed juvenile pink salmon (*Oncorhynchus gorbuscha*) showed an altered shape, reduced thickness of the compact myocardium, and hypertrophic changes in spongy, trabeculated myocardium, indicating that pathological remodeling occurred in juvenile pink salmon in response to crude oil exposure during embryonic cardiogenesis. In the study of Hicken et al. [39], transient embryonic exposure of zebrafish (*Danio rerio*) to very low concentrations of oil caused subtle changes in the ventricle shape (9% decrease in length-to-width ratio) of adult zebrafish, suggesting the legacy effect of crude oil in relation to cardiac toxicity. Similarly, though not as limited, our histopathological results also showed structural alteration in addition to morphological changes in the larvae heart. In reality, the developing heart is one of the first organs to become functional during organogenesis in fish. It is well established that cardiac function and morphology are inextricably interdependent processes that together shape heart development in fish and other vertebrates [40]. In this context, it has been shown that three-ring classes of polycyclic aromatic compounds (PACs) that are commonly enriched in crude oil (e.g., the tricyclic phenanthrene) can directly and specifically interfere with cardiac function [18]. From this point of view, the three-ring PAHs of concern were also detected in the crude oil samples of our study, which accounted for more than 50% of the Σ16PAHs and may play an important role in the process of the histopathological alteration of medaka larvae hearts. Importantly, it should be noted that these adverse anatomical changes are usually coupled with reduced cardiorespiratory performance such as lower critical swimming speeds, as observed in the studies of Hicken et al. [39] and Incardona et al. [41]. Therefore, for wild fish living in oiled habitats, if their larvae are acutely exposed to crude oils, an uncertain and long-lasting outcome still remains with respect to other functional impairments in surviving larvae, juveniles, or even adults resulting from cardiac structure lesions.

### 4.3. Developmental Toxicities

In this study, we examined marine medaka embryos and larvae during 21 d of exposure to explore the potential developmental toxicity of WAF to their early life stages. To the best of our knowledge, this study is the first to report the NOECs and LOECs of crude oils in the ecologically important marine fish, *O. melastigma* (McClelland, 1839), regarding its embryonic development, which is critical for a risk assessment of oil spill components in marine environment

Usually, it takes around an average of 14 d for marine medaka embryos to break the chorion and hatch into larvae, and their organogenesis process is basically completed at 11 dpf. The heart function of marine medaka embryos was assessed by heart rate in this study. In line with the histopathological lesions of the heart observed in medaka larvae in an acute test, we found that 60% of WAF significantly reduced the embryonic heart rate of marine medaka at 11 dpf, indicating that the blood supply of the heart was probably affected. Similarly, field-collected oil samples of the Deepwater Horizon oil spill occurring in the Northern Gulf of Mexico in 2010 resulted in deleterious cardiovascular effects, including irregular atrial arrhythmia and circulatory disruption culminating in pericardial edema in the early life stages of three fish species [42]. Additionally, Philibert et al. [17] found that the WAF of Macondo crude oil significantly decreased the heart rate of sheepshead minnow (*Cyprinodon variegatus*) embryos during a 9-d exposure period across both embryonic and larval stages. Moreover, heart rate decrease was also observed in polar cod (*Boreogadus saida*) after 37 d of exposure to WSF [6]. According to Gardner et al. [43], the structural and functional abnormalities of fish hearts caused by crude oils probably resulted from changes in the expression of genes involved in cardiomyocyte proliferation and hypertrophy, inflammation, and innate immunity, as observed in the juvenile pink salmon (*Oncorhynchus gorbuscha*). For marine medaka, an abnormal expression of genes associated with heart development was also thought to relate to the alteration of heart rate and cardiac abnormality after exposure to new pollutants such as perfluorooctane sulfonate and microplastics [44,45]. However, which genes are specifically related with the WAF of crude oils in marine medaka is still unclear. Considering that both the morphological and functional abnormalities were found in the early life stages of marine medaka in our study, the potential reasons behind this phenomenon should also be explored in terms of the gene expressions which are involved in fish heart development.

Hatching is known to be a crucial period during fish embryogenesis. The hatching success of fish embryos determines whether the fish population can be supplemented adequately, while hatching time can reflect whether the development process of fish embryos is affected by exposure to pollutants. Our results demonstrated that neither the total hatching success nor the hatching time were significantly affected by different concentrations of WAF, indicating that the hatching process of marine medaka was not influenced by the exposure scenario in this study. Similarly, in the study of Nahrgang et al. [6], the hatching rate of *Boreogadus saida* embryos showed no significant change after exposure to different concentrations of WSFs. Incardona et al. [18] also did not observe an effect on the hatching rate of *Clupea pallasi* embryos exposed to crude oil. In contrast, although the average hatching time of *Mallotus villosus* embryos was not affected after 32 d of exposure to WSF, the hatching rate decreased with increasing WSF concentrations [16]. Although the above studies with three marine fishes all adopted similar crude oil preparation methods as that in our study, the possible discrepancy of the toxic component concentrations of different crude oils in the exposure solutions, together with the different sensitivities of the three fish species, resulted in different reported results among the three studies. In this study, the WAF obtained from the crude oil using the selected preparation method was not enough to affect the hatching success and average hatching time of marine medaka embryos, the reason for which is possibly due to the adopting concentrations not reaching the threshold required to exert an influence.

To explore the teratogenic effect of WAFs, eight typical malformation symptoms during development, including pericardial edema, cardiac elongation, decreased blood circulation, yolk sac edema, craniofacial deformity, spinal curvature, body bleeding, and broken fins, were assessed on hatched medaka larvae after chronic exposure. However, none of the above malformation symptoms were observed in alive larvae. Nevertheless, this does not mean that the crude oil used in this study cannot result in developmental malformation to the early life stage of marine medaka. It should be noted that mortalities in the three concentrations of the chronic exposure groups ranged from 16.2% (15% WAF group) to 100% (60% WAF group) (Figure 2), and this may be due to the death of malformed individuals caused by continued exposure after hatching. Therefore, a more precise evaluation of the teratogenicity of crude oil immediately after hatching in marine medaka larvae is required. PAHs in crude oil are considered to be the main cause of developmental toxicity in fish at the early life stage [46], which can produce Blue Sac disease (BSD), with symptoms similar to those induced by dioxins [47]. These symptoms include body bleeding, pericardiac and yolk sac edema, cardiovascular diseases, craniofacial and spinal deformities, etc. [48]. In previous studies, various marine fishes have shown different degrees of BSD symptoms after exposure to WAF/WSF, with the most commonly found symptoms being spinal curvature, pericardial edema, and craniofacial deformity [18,33,35,49,50,51,52], indicating that the heart and skeleton are sensitive organs for fishes in their early life stages during development to the toxicity of crude oil. Although we did not observe skeletal development abnormalities in medaka embryos, we can confirm that the heart was the main target organ for the early life stages of marine medaka, as demonstrated by the histopathological lesions of larvae and a decrease in embryo heart rate.

## 5. Conclusions

Overall, in this study, the toxic effects of crude oil on the early life stages of marine medaka were investigated from the perspectives of mortality (embryos and larvae), histopathological lesions (larvae), as well as hatching and development (embryos). We found that, for all concentrations of WAF, there was no significant change in embryo hatching. However, the mortality of medaka larvae at the highest WAF exposure concentration was significantly increased for both acute and chronic exposures, indicating that oil spill which occurred in the Bohai Sea possibly had an adverse impact on marine fish populations. Moreover, the histopathological lesions of larvae and a decrease in embryo heart rate suggest that both the cardiac structure and the function of the early life stages of marine medaka can be influenced by either acute or chronic crude oil exposure. Our results provide a scientific reference for assessing the ecological risk of crude oil spills in marine environments.

## Figures and Tables

**Table 1:** Nominal concentrations of WAFs and measured concentrations of TPHs (*n* = 3).

Test Group	Nominal Concentrations of WAF, %	Measured Concentrations of TPHs, mg·L^−1^
Acute test	Control	0.00 ± 0.00
	9.53	0.62 ± 0.01
	17.15	0.65 ± 0.02
	30.86	1.80 ± 0.07
	55.56	3.27 ± 0.07
	100.00	6.14 ± 0.04
Chronic test	Control	0.00 ± 0.00
	15.00	1.12 ± 0.14
	30.00	2.07 ± 0.21
	60.00	3.39 ± 0.16

**Table 2:** Individual and total (Σ16PAHs) PAH concentrations analyzed in WAFs (*n* = 3).

PAHs	CAS No.	Aromatic Rings	Concentration, mg·L^−1^	Percentage, %
Naphthalene	91-20-3	2	0.21 ± 0.03	12.12
Acenaphthylene	208-96-8	3	0.20 ± 0.04	11.49
Acenaphthene	83-32-9	2	0.02 ± 0.00	1.32
Fluorene	86-73-7	3	0.29 ± 0.05	16.53
Phenathrene	85-01-8	3	0.14 ± 0.06	7.57
Anthracene	120-12-7	3	0.31 ± 0.16	16.66
Fluoranthene	206-44-0	4	0.04 ± 0.01	2.32
Pyrene	129-00-0	4	0.20 ± 0.03	11.49
Benzo(a)anthracene	56-55-3	4	n.d.	/
Chrysene	218-01-9	4	0.36 ± 0.03	20.48
Benzo(b)fluoranthene	205-99-2	5	n.d.	/
Benzo(k)fluoranthene	207-08-9	5	n.d.	/
Benzo(a)pyrene	50-32-8	5	n.d.	/
Dibenzo(a,h)anthracene	53-70-3	5	n.d.	/
Indeno(1,2,3-cd)pyrene	193-39-5	6	n.d.	/
Benzo(g,h,i)perylene	191-24-2	6	n.d.	/
Σ16PAHs	/		1.77	/

n.d. not detected due to less than the detection limit.

**Table 3:** Mortality rate of marine medaka embryos after 21 d of exposure in chronic test (*n* = 3).

Group	Control	WAF Concentration, %		
		15.00	30.00	60.00
Mortality, %	10.00 ± 10.00	23.33 ± 15.28	26.67 ± 28.87	26.67 ± 20.82

**Table 4:** NOECs and LOECs of different toxicity endpoints in chronic test.

Life Stage	Endpoints	NOECs	LOECs
		(WAF, %)	
Embryo	Mortality	60 ^1^	—
	Heart rate	30 ^2^	60 ^1^
	Hatching time	60 ^1^	—
	Percent hatching success (%)	60 ^1^	—
Larvae	Mortality	30 ^2^	60 ^1^
	Malformation percent (%)	60 ^1^	—

^1^ 2.07 mg·L^−1^ as TPHs; ^2^ 3.39 mg·L^−1^ as TPHs.

## Data Availability

Data are contained within the article.
